# Plant NLRs: Evolving with pathogen effectors and engineerable to improve resistance

**DOI:** 10.3389/fmicb.2022.1018504

**Published:** 2022-09-28

**Authors:** Biaoming Zhang, Mengting Liu, Yanchao Wang, Wenya Yuan, Haitao Zhang

**Affiliations:** State Key Laboratory of Biocatalysis and Enzyme Engineering, School of Life Sciences, Hubei University, Wuhan, China

**Keywords:** plant immunity, NLR, pathogen effector, resistosome, engineering, interaction

## Abstract

Pathogens are important threats to many plants throughout their lifetimes. Plants have developed different strategies to overcome them. In the plant immunity system, nucleotide-binding domain and leucine-rich repeat-containing proteins (NLRs) are the most common components. And recent studies have greatly expanded our understanding of how NLRs function in plants. In this review, we summarize the studies on the mechanism of NLRs in the processes of effector recognition, resistosome formation, and defense activation. Typical NLRs are divided into three groups according to the different domains at their N termini and function in interrelated ways in immunity. Atypical NLRs contain additional integrated domains (IDs), some of which directly interact with pathogen effectors. Plant NLRs evolve with pathogen effectors and exhibit specific recognition. Meanwhile, some NLRs have been successfully engineered to confer resistance to new pathogens based on accumulated studies. In summary, some pioneering processes have been obtained in NLR researches, though more questions arise as a result of the huge number of NLRs. However, with a broadened understanding of the mechanism, NLRs will be important components for engineering in plant resistance improvement.

## Introduction

A large number of microbes are pathogens for plants, which cause different kinds of diseases. However, plants have developed many strategies to acquire resistance to most of the pathogens after a long time of evolution. It is well known that there are two layers of innate immunity in plants. The first layer is composed of membrane-anchored receptors that can interact with small conserved molecules called pathogen-associated molecular patterns (PAMPs) or microbe-associated molecular patterns (MAMPs). Therefore, these receptors are named as pattern recognition receptors (PRRs) and initiate PAMP-triggered immunity (PTI) (Jones and Dangl, 2006). The level of PTI is usually weak and non-specific, which can easily be overcome by pathogen effectors (Jones and Dangl, 2006; Zhang and Wang, 2013). Comparatively, the second layer of plant immunity is controlled by special intracellular receptors that can recognize the effectors and trigger severe resistance, which is known as effector-triggered immunity (ETI) (Jones and Dangl, 2006; van Wersch et al., 2019).

Based on the phenotype after pathogen invasion, plant disease resistance can be classified as qualitative resistance and quantitative resistance. Genetically speaking, qualitative resistance is conferred by a single *resistance* (*R*) gene or a pair of related *R* genes, in a few cases, in plants. The *R* genes can confer resistance to pathogens that carry *avirulence* (*avr*) genes in the way known as the “gene for gene” hypothesis. Meanwhile, quantitative resistance is controlled by multiple different genes or loci in the genome. Usually, qualitative resistance is pathogen race-specific and can be even complete resistance, while quantitative resistance is pathogen race-nonspecific and partial resistance. Qualitative resistance is also simple and easy to manipulate. As a result, it is widely used in mechanism studies and crop improvement. To date, the number of cloned plant *R* genes is more than 300 (Kourelis and van der Hoorn, 2018). Although these *R* genes encode many different kinds of proteins, a large portion of the proteins belongs to nucleotide binding, leucine-rich repeat proteins (NLRs), which are also the most common intracellular receptors in ETI (Kourelis and van der Hoorn, 2018).

Plant NLRs harbor similar structural similarities that they all have an NB-ARC (nucleotide-binding adaptor, APAF-1, R proteins, and CED-4) domain and a leucine-rich repeat (LRR) domain. The N-termini of the typical NLRs are different, but only a few domains or motifs exist in this region, such as the Toll/interleukin receptor (TIR) domain, coiled-coil (CC) motif, or resistance to powdery mildew 8 (RPW8) domain. Based on these domains, the typical NLRs can be grouped into three groups, TIR-NLRs (TNLs), CC-NLRs (CNLs), and RPW8-NLRs (RNLs) (Maruta et al., 2022). Many TNLs and CNLs can interact with effectors directly or indirectly and are called “sensor” NLRs (sNLRs); by contrast, many RNLs function redundantly at downstream of TNLs and CNLs and are called “helper” NLRs (hNLRs) instead (Jubic et al., 2019). Intriguingly, not all hNLRs are RNLs. A special family of CNLs named as NRC (NB-LRR protein required for HR-associated cell death) family also belongs to hNLRs (Jubic et al., 2019).

However, there are still some atypical NLRs, which include additional integrated domains (IDs), such as WRKY, kinase, heavy metal-associated (HMA), and zinc-finger BED (zf-BED) domains (Brueggeman et al., 2008; Le Roux et al., 2015; Maqbool et al., 2015; Sarris et al., 2015; Marchal et al., 2018; Ji et al., 2020; Zhang et al., 2020). Some IDs interact with corresponding pathogen effectors, and thereby they are recognized as “integrated decoys” (Jones et al., 2016; Kroj et al., 2016). Such atypical NLRs are also named as NLRs with integrated domains (NLR-IDs), and many of them function together with other NLRs as NLR pairs (also called paired NLRs) (Jones et al., 2016). In this review, we summarize the research on the mechanism of NLRs in accordance with their classification. We then discuss about the recent attempts at NLR engineering, which brings us into a new research field.

## Most CNLs interact indirectly with effectors to confer resistance

The significant feature of CNLs is that most of them recognize the cognate effectors in an indirect manner. There are two models for describing this kind of recognition. The first is the “guard” model, in which the pathogen effectors interact with and modify their target proteins in plant cells, while the NLRs can monitor the integrity and modification of these target proteins. As a result, the NLRs are called “guarders” and the target proteins are “guardees”. Once the guardees changed their status, the NLRs trigger resistance in plants. The most well-known guardees are Arabidopsis RPM1-interacting protein 4 (RIN4) and PBS1, which are involved in the resistance mediated by many NLRs (Duxbury et al., 2021). The effectors AvrB and AvrRpm1 can target RIN4 and induce its phosphorylation to increase the activity of plasma membrane (PM) H^+^-ATPases and regulate the stomata re-opening (Liu et al., 2009). Then phosphorylated RIN4 activates the CNL RPM1 to confer resistance in Arabidopsis (Chung et al., 2011; Liu et al., 2011). The effector AvrRpt2 also targets and cleaves RIN4 into fragments to suppress PTI (Afzal et al., 2011). Whereas the Arabidopsis CNL RPS2 can interact with intact RIN4 and become active to trigger plant defense response after RIN4 is cleaved (Axtell and Staskawicz, 2003). Similarly, another CNL RPS5 interacts with and guards PBS1 in Arabidopsis. The effector AvrPphB can cleave PBS1, leading to the activation of RPS5 (Shao et al., 2003; Ade et al., 2007).

The second is the “decoy” model, which is a derivative and modification of the “guard” model. Compared with “guardees”, which usually are functional proteins and involved in defense response, many “decoys” barely have any significant function besides interacting with pathogen effectors (Jones et al., 2016; van Wersch et al., 2019). When the decoys are targeted and modified by the effectors, the NLRs recognize and interact with decoys to trigger defense reactions (Duxbury et al., 2021). One example is the Arabidopsis CNL ZAR1, which recognizes multiple decoys to confer resistance to diverse pathogens. The effector AvrAC of *Xanthomonas campestris* pathovar *campestris* uridylylates many proteins in Arabidopsis, such as BIK1 and RIPK, to attenuate their function in immunity (Feng et al., 2012). Whereas, PBL2, a homolog of BIK1, acts as a decoy and can be uridylylated by AvrAC, though it is not required for AvrAC virulence. And the uridylylated PBL2, PBL2^UMP^, can be recruited to the preformed complex of ZAR1 and RKS1 to trigger immunity (Wang et al., 2015). Nevertheless, it is difficult to make a distinction between “guardee” and “decoy” for certain proteins. Taking PBS1 as an example, although its homologs participate in PTI and also can be cleaved by the effector AvrPphB, PBS1 has the weakest function in PTI. As a result, PBS1 is also regarded as a “decoy” for AvrPphB (Zhang et al., 2010).

The sophisticated indirect interaction between CNLs and effectors is well-proved by the structure information of ZAR1. Before PBL2^UMP^ is recognized by the pre-active complex of ZAR1-RKS1, ZAR1 binds ADP through its NB domain and remains inactive. After the interaction between PBL2^UMP^ and RKS1, ADP is released and the conformation of the ZAR1 NB domain is changed (Wang et al., 2019b). With the exchange of ADP by dATP or ATP, the activated ZAR1-RKS1-PBL2^UMP^ complexes further oligomerize as a pentameric resistosome to confer resistance (Wang et al., 2019a). In contrast, the corresponding effector AvrAC is not a component in the ZAR1 resistosome.

Meanwhile, a few CNLs have been found to interact directly with pathogen effectors or PAMPs. In tomatoes, a Solanaceae domain (SD) exists at the terminus of the CNL Sw-5. The Sw-5 SD physically interacts with a conserved region in the viral movement protein NSm from tospoviruses and activates Sw-5 to initiate a defense response (Li et al., 2019). In barley, proteins encoded by several alleles of the *Mla* gene can interact with many natural AVR_A_ effectors of barley powdery mildew pathogen in tobacco (Saur et al., 2019).

**Figure 1:**
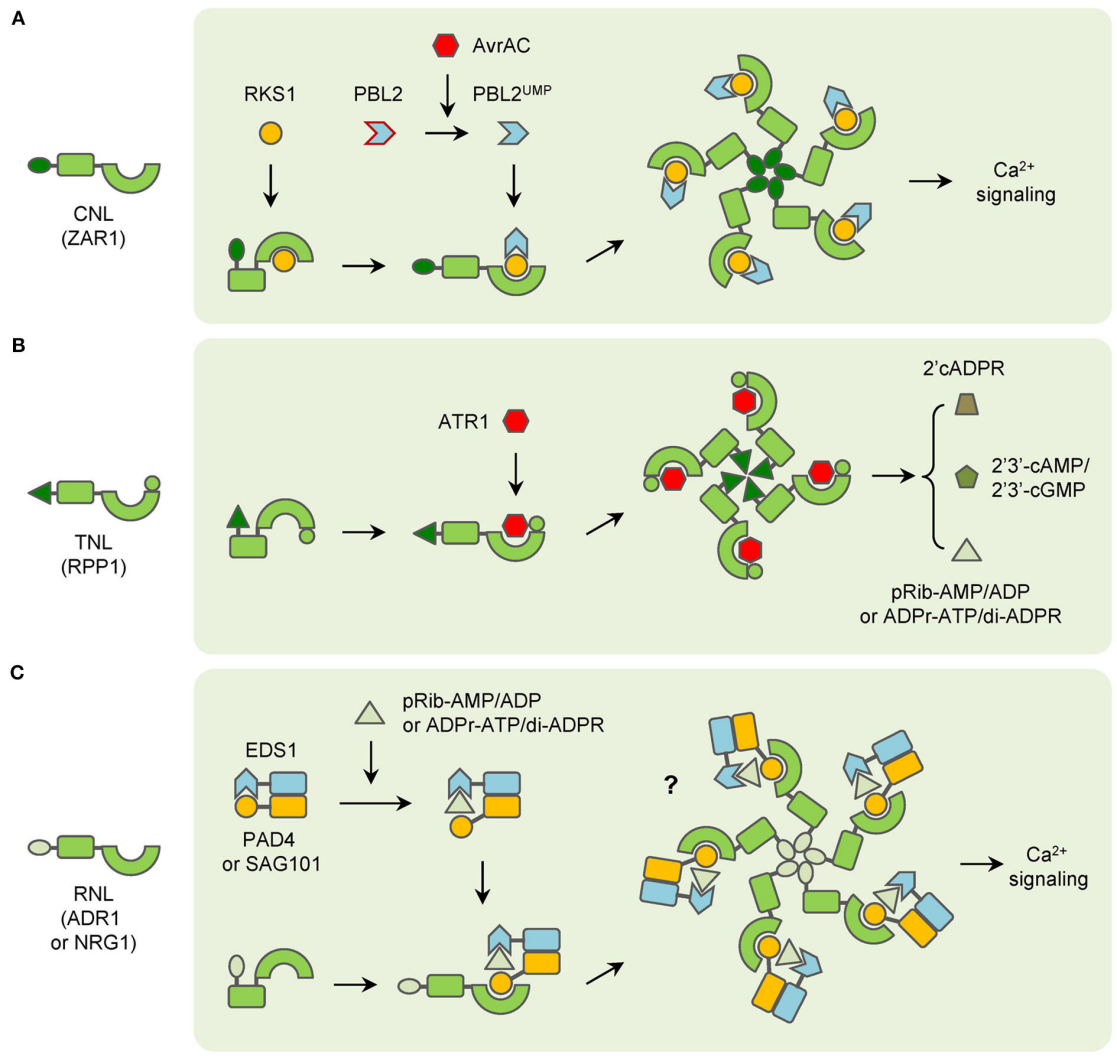
Functioning models of three groups of typical NLRs. **(A)** CNLs (ZAR1 as an example) recognize pathogen effectors indirectly and form resistosomes with associated proteins to trigger immunity. **(B)** TNLs (RPP1 as an example) directly interact with pathogen effectors and then form resistosomes in plants immunity. **(C)** RNLs (ADR1 and NRG1 as examples) function with EDS1 complexes, which are activated by TNLs-generated small molecules to trigger immunity.

The precise function of CNLs has been studied in plants. However, the results are different and some kind of controversial from each other. In barley, the CC domain alone is sufficient to induce cell death for MAL10 (Maekawa et al., 2011; Bai et al., 2012). And self-association of the CC domain is found to be essential for MAL10-triggered immunity (Maekawa et al., 2011). In potatoes, the Rx protein triggers a hypersensitive response (HR) through its NB domain instead of the CC domain (Rairdan et al., 2008). Whereas in Arabidopsis, both the CC and NB-ARC domains are required for HR-inducing of RPS5 (Qi et al., 2012). After the discovery of the ZAR1 resistosome, the CC domain is recognized as the key element for the CNL resistosome. The CC domains formed a helical barrel in the ZAR1 resistosome, and the biochemical function of the whole resistosome has been clarified as a cation-selective channel permeable to calcium ion (Ca^2+^) last year (Wang et al., 2019a; Bi et al., 2021). When ZAR1 resistosome is activated, it can be localized into the PM and cause Ca^2+^ influx, which results in perturbation of organelles structures, induction of reactive oxygen species (ROS), and cell death in plants (Figure 1A; Bi et al., 2021). Likewise, the Ca^2+^ influx is also increased in RPM1-mediated resistance and leads to hydrogen peroxide (H_2_O_2_) accumulation and HR in Arabidopsis (Grant et al., 2000). However, whether other CNLs, such as Rx, function in the same way still remains unclear now.

## Many TNLs directly recognize effectors to confer resistance

The TIR domain is a well-known structure that can be found not only in plants but also in animals and bacteria. Moreover, TIR domains are critical components of innate immune proteins in both plants and animals (Ve et al., 2015). Unlike that TIR domains occur in many structurally different proteins in animals, they mainly exist in TNLs, TIR-NB (TN) proteins, which lack LRRs, and TIR-only (or named as TX) proteins in plants (Nandety et al., 2013; Ve et al., 2015). The TIR domains are involved in protein–protein interactions, including self-association. In plants, overexpression of TIR regions of several TNLs can trigger HR in an effector-independent manner. Mutations disrupting the TIR self-association affect its auto-activation and the HR reaction (Bernoux et al., 2011). Moreover, chimeric NLRs that are constructed from TIR domains of plant TNLs and other domains of mammalian NLRC4 can trigger HR in plants when co-expressed with other inflammasome components (Duxbury et al., 2020). As a result, the induced proximity of TIR domains is crucial for plant defense (Duxbury et al., 2021).

However, the function of TIR is influenced by other domains in TNLs. It has been reported that the TIR-triggered HR can be inhibited by NB-ARC domains (Bernoux et al., 2011; Schreiber et al., 2016). Cryo-electron microscopy (cryo-EM) structure information has revealed that TNLs, like tobacco ROQ1, bind ADP to keep NB-ARC domains in a closed state and inhibit self-association (Martin et al., 2020; Maruta et al., 2022). An exception is Arabidopsis RPP1, which binds ATP instead of ADP and maintains inactive by NB-ARC associated with LRR (Ma et al., 2020). Nevertheless, after the cognate effectors are recognized through the C-JIDs (C-terminal jelly roll/Ig-like domains) and LRR domains, conformations of NB-ARC domains are changed and TNLs assemble as polymers, which leads to the proximity of TIRs to trigger immunity (Ma et al., 2020; Martin et al., 2020).

Though the LRR domains participate in effector recognition, C-JIDs are the key determinants. Many studies have also revealed that truncation, deletion, or mutation in the C-JIDs impairs TNL-mediated immunity (Dodds et al., 2001; Saucet et al., 2021). Some TNLs even contain more than one C-JIDs. The *Ma* gene in Myrobalan plum encodes a TNL with five C-JIDs located at the C-terminus and confers complete-spectrum and high-level resistance to root-knot nematode (RKN) (Claverie et al., 2011; Maruta et al., 2022). While Arabidopsis RPS4, which forms an NLR pair with RPP1, is an exception (Birker et al., 2009; Narusaka et al., 2009). The C-JID of RPS4 is involved in the maintenance of its inactive state and cannot interact with the corresponding effectors (Saucet et al., 2021). In addition, the TNL N protein recognizes the 50 kDa helicase (p50) effector of the *Tobacco mosaic virus* (TMV) through its TIR domain in an indirect manner. The N receptor-interacting protein 1 (NRIP1) interacts with both N and p50 and forms a complex in plant defense response (Caplan et al., 2008).

The biochemical function of TIR domains was unknown for a long time until it was first characterized in human protein SARM1 (sterile alpha and TIR motif containing 1), which belonged to Toll-like receptor (TLR) proteins. The SARM1-TIR domain has intrinsic NADase (nicotinamide adenine dinucleotide nucleosidase) activity and contains an essential glutamic acid residue (Essuman et al., 2017). Recently, TIR domains of many plant TNLs have been found to possess NAD^+^ cleavage activity and produce cyclic adenosine diphosphate ribose (cADPR), cyclization variant of cADPR (v-cADPR), and nicotinamide (NAM) (Wan et al., 2019). Notably, the NADase activity is dependent on the self-association of TIR domains in both animals and plants (Horsefield et al., 2019; Wan et al., 2019). The self-association has also been confirmed by the cryo-EM structures of RPP1 and ROQ1. After interacting with corresponding effectors, both TNLs assemble as tetrameric resistosomes. In each resistosome, two asymmetric TIR homodimers form a holoenzyme with two active sites at the groove in the homodimer (Ma et al., 2020; Martin et al., 2020). However, the characterization of chimeric NLRC4 that fused with plant and animal TIR domains has shown that the NADase activity is essential but not sufficient for plant defense reaction (Duxbury et al., 2020). And recently, it has been found that the plant TIR domains are bifunctional enzymes, which also synthesize 2',3'-cAMP/cGMP with DNA or RNA and lead to cell death (Yu et al., 2022).

In brief, for many TNLs, the NB-ARC and LRR domains stay in a closed state and inhibit the self-association of TIR domains to keep TNLs inactive without the presence of cognate effectors. When the effectors appear, they can interact with the C-JIDs and LRR domains of TNLs. After recognition, the conformation of NB-ARC domains is changed and they participate in self-association and lead to the oligomerization of TNLs. Then the assembly of activated TNLs induces the proximity of TIR domains to make up the holoenzymes and generate certain molecules. The molecules of the resistosomes then transduce the signaling to other downstream components in plant immunity (Figure 1B).

## Most RNLs are crucial downstream components of NLR signaling

There are two families of RNLs, the ADR1 (ACTIVATED DISEASE RESISTANCE 1) family and the NRG1 (N REQUIREMENT GENE 1) family (Duxbury et al., 2021). Neither of the two families is involved in the recognition of pathogen effectors. Instead, they function at the downstream of CNLs and TNLs (Jubic et al., 2019). Some TNLs prefer the ADR1 family to transduce signaling in immunity. For example, a triple mutant of the *ADR1* gene family fully suppresses the gain-of-function mutant *snc1*-mediated auto-immunity in Arabidopsis, which indicates that ADR1 family proteins function redundantly downstream of the TNL SNC1 (Dong et al., 2016). Similar results have been found for RPP2 and RRS1 (Saile et al., 2020). While most of the TNLs prefer the NRG1 family in signaling transduction. Mutants of the *NRG1* family lead to impaired HR in tobacco and Arabidopsis which express different TNLs (Castel et al., 2019). Interestingly, the resistance mediated by the TNL WRR4A is attenuated when both *ADR1* and *NRG1* gene families are mutated in Arabidopsis, which suggests that ADR1 and NRG1 family proteins are fully redundant in WRR4A-conferred resistance (Saile et al., 2020). Similarly, the requirement of ADR1 family proteins has also been found in some CNLs-mediated immunity (Bonardi et al., 2011).

Another important constituent of NLR signaling is the EDS1 (enhanced disease susceptibility 1) family, which contains EDS1, PAD4 (phytoalexin deficient 4), and SAG101 (senescence-associated gene 101) (Duxbury et al., 2021; Maruta et al., 2022). Though these proteins contain N-terminal lipase-like domains, they do not possess catalytic activity. In fact, EDS1 can form heterodimers with either PAD4 or SAG101 (Wagner et al., 2013). The EDS1-PAD4 and EDS1-SAG101 dimers are involved in both PTI and ETI, especially in TNL signaling (Dongus and Parker, 2021).

Recent studies have found that RNLs and EDS1 family proteins constitute complexes to regulate TNL signaling. In Arabidopsis, EDS1 and SAG101 interact with NRG1, not ADR1 after the effector XopQ activate ROQ1-triggered immunity (Lapin et al., 2019; Sun et al., 2021). A member of the ADR1 family, ADR1-L1, interacts with EDS1-PAD4 heterodimer in TX protein RBA1-triggered immunity, and the interaction is reduced if the NADase activity is mutated in RBA1 (Wu et al., 2021). As a result, the molecules produced by TIR domains are expected to transduce the signals from activated TNLs to downstream EDS1-PAD4 and EDS1-SAG101 complexes, like second messengers. However, it remains unsure what the product is until two groups of compounds are reported very recently. The 2′-(5″-phosphoribosyl)- 5′-adenosine mono-/di-phosphate (pRib-AMP/ADP) are produced after RBA1 is activated. They bind to the EDS1-PAD4 complex and lead to conformational changes, which promote the interaction with ADR1 in immunity (Huang et al., 2022). Similarly, ADP-ribosylated ATP (ADPr-ATP) and ADPr-ADPR (di-ADPR) are generated by TIR domains of RPP1 and RPS4. They can bind to the EDS1-SAG101 complex and induce the interaction with NRG1A (also named as NRG1.1), a member of the NRG1 family (Jia et al., 2022).

Notably, the RPW8 domains at the N-terminus of RNLs belong to an ancient class of CC domains that are also called the CC-R domain. The CC-R domains are homologous to the 4HB domain in animal MLKL (mixed lineage kinase domain like), which causes a rapid influx of Ca^2+^ and necroptotic cell death after being activated (Gong et al., 2017; Jubic et al., 2019). After the structure of the NRG1.1 CC-R domain was discovered, it has been found that the CC-R domain resembles the four-helical bundle of the ZAR1 CC domain. And both NRG1.1 and ADR1 have been found to form Ca^2+^-permeable channels in plants (Jacob et al., 2021). In addition, ADR1 and NRG1 family members trigger auto-immunity if the MHD motifs are modified (Roberts et al., 2013; Wu et al., 2019). Nonetheless, whether EDS1-PAD4-ADR1 or EDS1-SAG101-NRG1 complex would form a resistosome similar to ZAR1 in plant immunity is still not confirmed. In summary, many RNLs possess the same biochemical function as CNLs, but the function is regulated by the interaction with EDS1-PAD4 or EDS1-SAG101 heterodimers after the second messengers are derived by pathogen effector-activated TNLs (Figure 1C). However, a report has shown that overexpression of NRG1 alone can trigger resistance to HopQ1-carrying *Pseudomonas syringae* pv. *tomato* (*Pto*) DC3000 in Arabidopsis. Given that the TNL-encoding gene *Roq1* does not exist in the Arabidopsis genome, NRG1 is considered to recognize the effector HopQ1 directly (Brendolise et al., 2018). This report has indicated that RNLs may have additional functions in plant immunity. Meanwhile, the mechanism of RNLs function in CNL-mediated immunity is still largely unknown.

## Atypical NLRs confer resistance in diverse manners

**Figure 2:**
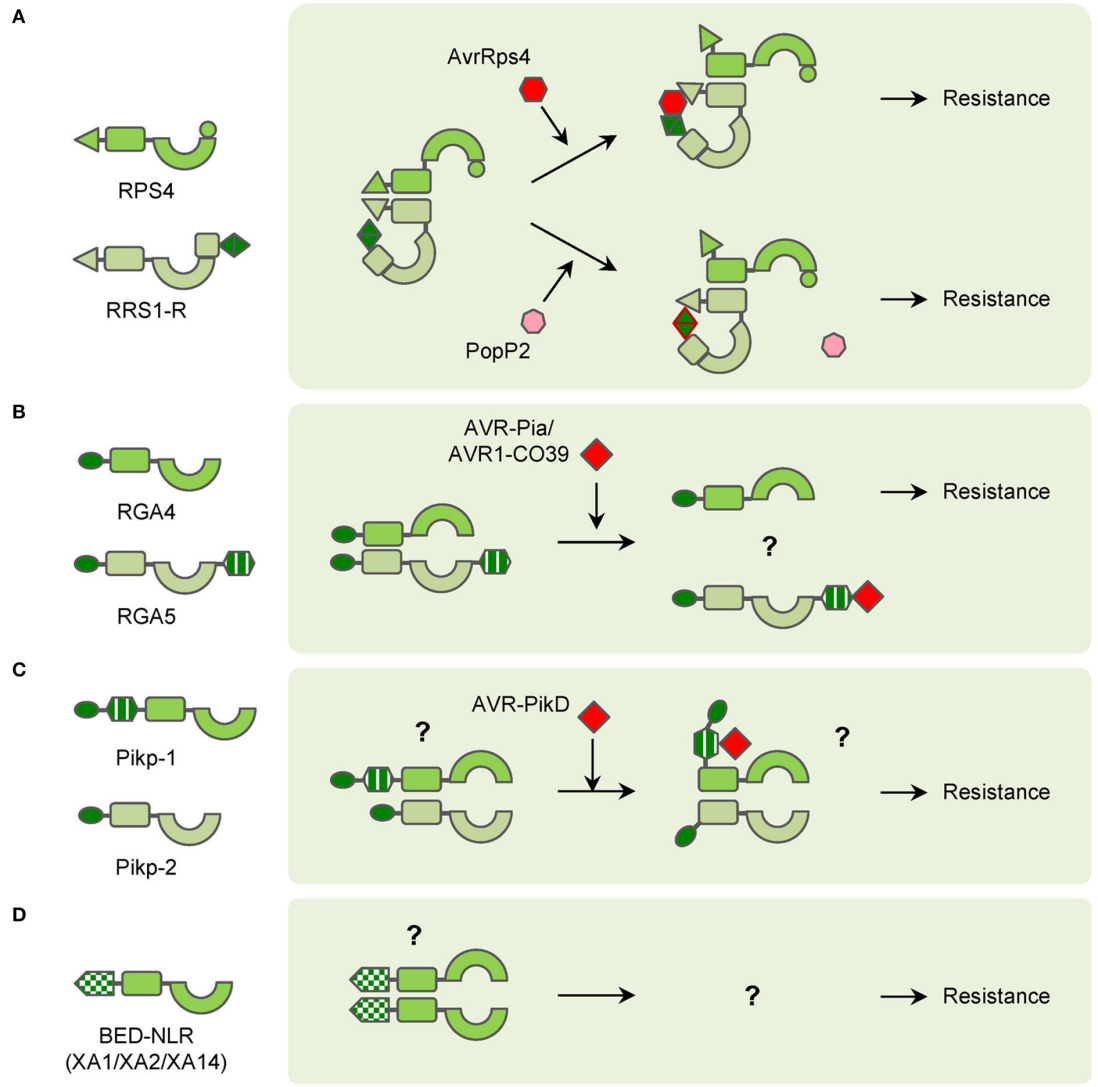
Diagrams of interactions between pathogen effectors and atypical NLRs in NLR pairs or singletons. **(A)** RPS4/PPS1-R NLR pair functions in different ways with pathogen effectors AvrRps4 and PopP2 to confer resistance. **(B)** RGA4/RGA5 NLR pair recognizes pathogen effectors AVR-Pia and AVR1-CO39 through the RGA5 HMA domain to confer resistance. **(C)** Pikp-1/Pipk-2 NLR pair interacts with pathogen effector AVR-PikD through Pikp-1 HMA domain to confer resistance. **(D)** BED-NLRs (XA1/XA2/XA14 as examples) probably form polymers to confer resistance.

Compared with classical NLRs, many atypical NLRs have extra domains or motifs, which also play important roles in plant immunity. A large portion of atypical NLRs are members in NLR pairs. In Arabidopsis, RPS4 and RRS1 function as an NLR pair to confer resistance to PopP2 from *Ralstonia solanacearum* and AvrRps4 from *Pseudomonas syringae* pv. *pisi* (Narusaka et al., 2009). Structurally, RPS4 is a common TNL while RRS1 contains an additional WRKY domain at the C-terminus. TIR domains of RPS4 can form homodimers and induce HR. However, the HR can be abolished by the RRS1 TIR domain because the TIR domains of RRS1 and RPS4 form a more stable heterodimeric complex (Williams et al., 2014). Though overexpression of *RPS4* induces weak HR, *RPS4* autoactive alleles, which contain mutations in the NB-ARC domain cause increased HR in the presence of RRS1-R1. These results strongly suggest that RRS1 plays a sophisticated role in regulating RPS4-mediated immunity (Guo et al., 2021). The effector PopP2 possesses acetyltransferase activities and acetylates many WRKY transcription factors (TFs), which leads to a reduction of WRKY-DNA interaction and suppression of PTI in Arabidopsis. Meanwhile, RRS1-R1 uses the WRKY domain as an ‘integrated decoy' to detect the appearance of PopP2, and its acetylation further activates the RRS1-R/RPS4 complex (Le Roux et al., 2015; Sarris et al., 2015). The effector AvrRps4 is hydrolyzed into two fragments after entering the plant cell and the C-terminal fragment (AvrRps4^C^) can interact with the WRKY domain of RRS1-R1 (Sohn et al., 2009, 2012; Mukhi et al., 2021). Without AvrRps4, the WRKY domain of RRS1 interacts with the domain 4 (DOM4) and results in the inactive (pre-activation) state of the RRS1/RPS4 complex (Ma et al., 2018; Guo et al., 2020). After recognition of AvrRps4 by RRS1 WRKY domain, the interaction between RRS1 TIR and its C-terminus is enhanced, which releases the RPS4 TIR from the heterodimer with RRS1 TIR (Sarris et al., 2015; Guo et al., 2020). In summary, RRS1 is a ‘sensor NLR' that detects effectors *via* its WRKY domain, the RPS4 is an ‘executor NLR' that triggers plant immunity after being activated. In the pre-activation state, RRS1 and RPS4 form the heterodimer, and RRS1 TIR inhibits the activity of RPS4 TIR. The effectors can interact with or modify the RRS1 WRKY domain to enhance the proximity of RRS1 TIR to its C terminus. As a result, the RPS4 TIR is released and initiates plant immunity (Figure 2A).

Meanwhile, in rice, many NLRs use heavy metal associated (HMA) domains as integrated decoys. *Pia*-mediated resistance to rice blast is conferred by the CNL pair RGA4/RGA5 (Okuyama et al., 2011). RGA5 contains an HMA domain at the C-terminus, which interacts with the effectors AVR1-CO39 and AVR-Pia of the fungus *Magnaporthe oryzae* (Cesari et al., 2013). RGA4 acts as an executor NLR, which can induce strong HR in tobacco leaves. RGA5 interacts with RGA4 and represses the HR when co-expressed in the absence of cognate effectors. And if AVR-Pia is co-expressed additionally, the HR can be resumed because of the recognition by the RGA5 HMA domain (Figure 2B; Cesari et al., 2014). The HMA domains also exist in NLR pairs encoded by *Pik* and its alleles, such as *Pikm* and *Pikp* (Ashikawa et al., 2008; Yuan et al., 2011). The HMA domains exist between the CC and NB-ARC domains in Pikp-1 and Pikm-1 and interact with AVR-Pik variants (Maqbool et al., 2015; De la Concepcion et al., 2018). Unlike RGA4, Pikp-1 or Pikp-2 alone cannot induce HR in tobacco. However, co-expression of Pikp-1, Pikp-2, and their corresponding effector AVR-PikD induces significant HR in tobacco (Figure 2C; Maqbool et al., 2015). Pikm-1 has variations in the HMA domain and binds more effectors than Pikp-1 (De la Concepcion et al., 2018). AVR-Pia, AVR1-CO39, and AVR-PikD are different in sequence but similar in structure and all belong to the MAX (*Magnaporthe* Avrs and ToxB like) effector family (de Guillen et al., 2015). The structural similarity among the MAX effectors also leads to the discovery that *Pikp* confers partial resistance to *M*. *oryzae* expressing AVR-Pia, which is caused by the “mis-matched” interaction between AVR-Pia and Pikp-1 HMA domain (Varden et al., 2019). Intriguingly, although AVR-Pia (or AVR1-CO39) and AVR-PikD are recognized by the HMA domains, the interactions occur at opposite surfaces of HMA domains, which is possibly the result of convergent evolution for rice to cope with varied pathogen effectors (Guo et al., 2018; Varden et al., 2019).

In a few pieces of literature, the “executor NLR” is also called “helper NLR” (Adachi et al., 2019; De la Concepcion et al., 2019). However, this might lead to confusion with RNLs and NRCs in some cases. In most NLR pairs, the two NLRs often interact with each other in the recognition of pathogen effectors. And NLRs such as RPS4 and RGA4 are capable to induce HR alone, instead of functioning at the downstream of the sensor NLRs. Based on these features, we prefer to group them into “executor NLRs” here. To further distinguish helper NLR and executor NLR in NLR pairs, we refer readers to recent reviews (van Wersch et al., 2019; Sun et al., 2020).

Besides participating in NLR pairs, a few atypical NLRs function as “singletons” without partner NLRs. Due to the lack of knowledge, the function of their IDs is unknown yet. The zf-BED is commonly found in chromatin-associated proteins and transposases (Aravind, 2000). Nevertheless, several NLRs in rice and wheat contain zf-BED domains for resistance to different pathogens. In wheat, *Yr5, Yr7*, and *YrSP* are three allelic yellow stripe rust *R* genes. The zf-BED domains are located at the N-terminus of the Yr5, Yr7, and YrSP proteins. Mutation in the zf-BED domain of Yr7 attenuates its resistance, though the mechanism is unclear (Marchal et al., 2018). In rice, *Xa1, Xa2, Xa14*, and *Xo1* are all allelic *R* genes for resistance to *Xanthomonas oryzae* pv. *oryzae* (*Xoo*) and *Xanthomonas oryzae* pv. *oryzicola* (*Xoc*) (Yoshimura et al., 1998; Ji et al., 2020, Read et al., 2020; Zhang et al., 2020). The proteins encoded by these genes are homologous to Yr5 and Yr7, and they also harbor zf-BED domains at N-terminus (Ji et al., 2020; Zhang et al., 2020). The zf-BED domains of XA1, XA2, and XA14 interact with themselves and also one another, which indicates homodimers and heterodimers may be formed in the resistance process (Figure 2D; Zhang et al., 2020). It has been reported that many transcription activator-like effectors (TALEs) are crucial for *Xa1*-mediated resistance, but the physical interaction between them is still uncovered yet (Zhao et al., 2016; Zhang et al., 2022).

## NLRs can be engineered for improved resistance

**Figure 3:**
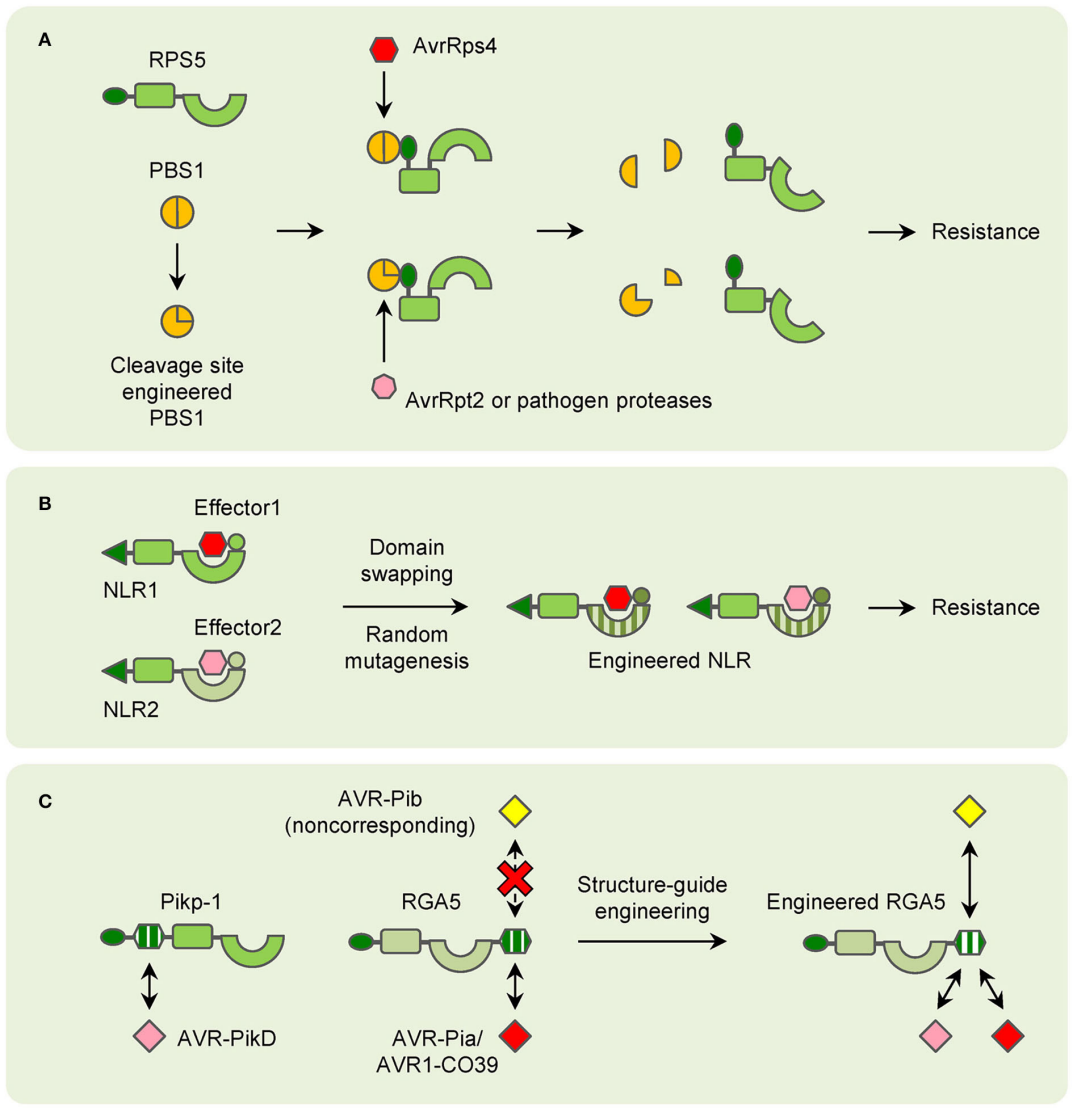
Models of NLR engineering in plants. **(A)** Modification of decoy proteins (PBS1 as an example) to confer resistance to new pathogen effectors. **(B)** Domain-swapping and random mutagenesis of NLRs to enlarge the resistance spectrums. **(C)** Structure-guide engineering of NLRs to confer resistance to noncorresponding effectors.

After a long time of evolution, plants have evolved many strategies to cope with various pathogens. For NLRs that recognize effectors indirectly, the guardees or decoys are the determinants of their resistance. RIN4 is targeted by lots of bacterial effectors to disturb plant PTI, and many NLRs guard RIN4 for triggering ETI for resistance (Ray et al., 2019). Besides RKS1, ZAR1 can interact with many other different ZAR1-ASSOCIATED KINASEs (ZRKs), which probably function as sensors for diverse effectors (Liang and Zhou, 2018). As a result, modification of the guardees or decoys can lead to changes in pathogen recognition in theory. In the past few years, this has been proved by the engineering of PBS1. When the AvrPphB cleavage site is replaced by sites for other pathogen protease, PBS1 confers resistance to new pathogens (Kim et al., 2016). Such a strategy is also applicable to crops. Modification of the PBS1 ortholog in soybean can lead to significant resistance to soybean mosaic virus (SMV) (Figure 3A; Pottinger et al., 2020).

In contrast, for NLRs that directly interact with pathogen effectors, duplication and recombination of their encoding genes for generating orthologs and paralogs are easily found in most plant genomes. Thus, many NLR-encoding genes are allelic and mediate resistance with specific spectrums. It is easy to understand that the variations in the domains which are involved in pathogen effector recognition are key for the resistance specificity. It has also been found for a long time that mutation or changes in this region can lead to the changement in resistance (Ellis et al., 1999; Dodds et al., 2001). And domain-swapping has been used in many studies for generating new NLRs for a long time. Taking the flax *L* locus as an example, it contains several allelic genes that confer resistance to different strains of flax rust fungus *Melampsora lini*, which carry different effectors (Ellis et al., 1999). A recombinant chimeric protein based on L5 and L6 has exhibited a novel and expanded spectrum in tobacco (Figure 3B; Ravensdale et al., 2012). Random mutagenesis is another strategy to select synthetic NLRs with expanded spectrums (Harris et al., 2013; Segretin et al., 2014). In potatoes, the CNL R3a confers resistance to the late blight pathogen *Phytophthora infestans* (*P. infestans*). R3a recognizes the effector AVR3a of *P. infestans* but responds weakly to its allele AVR3a^EM^. A random mutant library has been generated and many clones with expanded responses to AVR3a^EM^ have been obtained (Figure 3B; Segretin et al., 2014). However, there are still disadvantages to domain-swapping and random mutagenesis. The efficiency and effectiveness are unpredictable until the chimeras or mutants are tested. Usually, plenty of recombinants should be employed to fully cover the whole variations, and only a small number of them could meet the objectives. And due to the limitation of template sequences, new spectrums are commonly limited within the species which the templet NLRs confer resistance to.

Recently, with the application of structural biology technology, detailed interactions between certain NLRs and pathogen effectors have been uncovered. These results have greatly facilitated the attempts of NLR engineering. The most well-known examples are the modification of NLRs for rice blast resistance. The structure of the AVR-PikD bound Pikp-1 HMA domain has been used in screening mutations of Pikp-1 for expanded effector recognition, and one mutation has displayed increased affinity to many AVR-Pik variants in tobacco (De la Concepcion et al., 2019). Because of the conservation of HMA domains, RGA5 has been engineered to recognize AVR-PikD based on structural information of the Pikp-1_HMA/AVR-PikD complex without affecting AVR-Pia recognition. The engineered RGA5 indeed interacts with both AVR-Pia and AVR-PikD in tobacco, while it only confers resistance to *M*. *oryzae* isolates carrying AVR-Pia in rice (Cesari et al., 2022). Furthermore, the HMA domain of RGA5 has been modified for interacting with the noncorresponding effectors. Though the *M*. *oryzae* effector AvrPib initiates *Pib*- not *Pia*-mediated resistance, it belongs to the MAX family and folds into a structure similar to AVR1-CO39. As a result, structure-guided engineering of the RGA5 HMA domain is carried out and a designed RGA5-HMA2 domain that interacts with AvrPib instead of AVR1-CO39 is found. Remarkably, the designed NLR RGA5^HMA2^, which carries the RGA5-HMA2 domain, confers significant resistance to *M*. *oryzae* isolates carrying AVR-Pib in the presence of RGA4 in rice (Figure 3C; Liu et al., 2021). All these attempts have shed light on the future that NLRs can be designed as predicted for improved plant immunity.

## Future perspectives and challenges

The researches on NLRs have obtained great progress in recent years, especially in the discoveries and functional characterizations of NLR resistosomes, as well as the interactions between different IDs and their cognate effectors. However, the number of NLRs in plants is plentiful and most of them are still unclear or uncovered yet. Even for the well-characterized ZAR1, whether it forms the same resistosome in resistance to pathogens carrying other effectors, such as HopZ1a, HopF2, HopBA1, HopO1, and HopX1, is still unknown (Martel et al., 2020). Lately, the CNL PigmR has been reported to guard the deubiquitinase PICI1 (PigmR-interacting and chitin-induced protein 1) from being degraded by *M*. *oryzae* effector Avr-Pi9 to initiate immunity in rice (Zhai et al., 2022). The interaction occurs between the CC domain of PigmR and PICI1, and this raises the question of detailed complex composition in the possible resistosome. RNLs function at the downstream of both PTI and ETI, and more studies may be carried out according to the existing results for each RNL. Current researches have shown that the relationship between PTI and ETI is more complicated than expected. Some NLR-mediated ETI responses have been reported to enhance ROS production and key components of PTI signaling in Arabidopsis (Ngou et al., 2021). Meanwhile, PRRs mutants have been found to be impaired in ETI response mediated by certain NLRs (Ngou et al., 2021, (Yuan et al., 2021)). These data raise the possibility that the PTI and ETI pathways mutually potentiate plant immunity. However, whether similar results could be found for all the NLRs are unsure. Furthermore, the whole structures of atypical NLRs are also important questions and should be different from each other. In addition, whether the IDs in some atypical NLRs, such as BED-NLRs, function as decoys is still uncertain, which should be illustrated first.

Recently, AvrPiz-t, another MAX effector from rice blast fungus, has been found to suppress rice immunity by exploiting ROD1 (RESISTANCE OF RICE TO DISEASES1) (Gao et al., 2021). AvrPiz-t also has the conserved structure of MAX effectors, and whether an engineered RGA5 or Pikp-1, which recognizes AvrPiz-t, could be designed is an intriguing question. Though still there are many questions to be fixed, the attempts at NLR engineering have already pointed out the direction in the future. The difficulties in studying an NLR structure are its high molecular weight and polymerization, and they could be solved with the help of developed cryo-EM technology. In general, with the accumulation of researches on the characterization and engineering of NLRs, designed plant immunity will be possible and benefit crop production in the future.

## Author contributions

HZ conceived the manuscript. BZ, ML, and YW prepared the original draft and figures. HZ and WY reviewed and edited the manuscript. All authors have read and approved the final manuscript.

## Funding

This work was supported by grants from the National Natural Science Foundation of China (32101747 and 31872811).

## Conflict of interest

The authors declare that the research was conducted in the absence of any commercial or financial relationships that could be construed as a potential conflict of interest.

## Publisher's note

All claims expressed in this article are solely those of the authors and do not necessarily represent those of their affiliated organizations, or those of the publisher, the editors and the reviewers. Any product that may be evaluated in this article, or claim that may be made by its manufacturer, is not guaranteed or endorsed by the publisher.
